# A Case Report of a Collodion Baby: An Autosomal Recessive Genodermatosis

**DOI:** 10.7759/cureus.37418

**Published:** 2023-04-11

**Authors:** Sabiha Quazi, Adarshlata Singh, Khalid Khan, Umesh Biyani

**Affiliations:** 1 Department of Dermatology, Venereology, and Leprosy, Jawaharlal Nehru Medical College, Datta Meghe Institute of Medical Sciences, Wardha, IND; 2 Department of Medicine, Jawaharlal Nehru Medical College, Datta Meghe Institute of Medical Sciences, Wardha, IND; 3 Department of Pediatrics, Kalpataru Clinic and Diagnostic Center, Nagpur, IND

**Keywords:** disorders of cornification, prenatal invasive tests, lamellar ichthyosis, genodermatoses, collodion baby, congenital ichthyosis

## Abstract

Congenital ichthyosis refers to various underlying genodermatoses that indicate prenatal epidermal abnormalities. Collodion babies are manifestations of rare congenital ichthyosis, comprising severe clinical complications that contribute to the risk of mortality. This case report presents the case of a full-term female neonate, delivered at 38 weeks of gestation, who exhibited features of a translucent collodion membrane over her entire body at birth. The mother reported fewer antenatal check-ups and a lack of obstetric ultrasonography during pregnancy. The baby later developed systemic complications, which were managed with intensive neonatal care. This case report attempts to address the uncommon occurrence of collodion babies, which can be managed with supportive care and diagnosed with a fair amount of certainty with invasive prenatal diagnostics.

## Introduction

Collodion baby (CB) is a rare form of congenital ichthyosis characterized by hyperkeratosis, severe erythroderma, and a slight desquamation pattern. This uncommon clinical condition has an estimated prevalence of one in 300,000 live births [1]. These neonates are born preterm and are covered in collodion. Collodion is a translucent skin covering that resembles parchment paper and peels off within the first two to four weeks of life, to exhibit a variety of congenital diseases with cutaneous and articular symptoms [2]. Trichothiodystrophy of the entire trunk and limbs, ectropion (eversion of the eyelids), eclabium (eversion of the lips), and hyperplasia of the nasal bones, auricular pavilions, fingers, and toes are characteristic manifestations of CB.

It eventually manifests as a multitude of abnormalities, including autosomal recessive congenital ichthyosis (ARCI), lamellar ichthyosis (LI), Harlequin ichthyosis, and non-bullous congenital ichthyosiform erythroderma (NBCIE). The tight membrane present at birth acts as a cause of certain complications in CBs, including the limited movement of the extremities and digits owing to pseudo-contractures, constricted chest expansion, scanty body hair, electrolyte imbalance, irregular body temperature, and malformed nose and ears are all symptoms of poor skin barrier function [3].

Therefore, a multidisciplinary treatment approach involving dermatologists and pediatric surgeons should be used to optimize the likelihood of long-term survival.

## Case presentation

A full-term female newborn with a glossy and translucent membrane all over her body since birth was admitted in the neonatal intensive care unit (NICU). The baby was born through non-consanguineous marriage to a 24-year-old mother with no relevant history of a similar dermatological condition in the family. The baby was delivered at 38 weeks of gestation by active labor and she weighed 2.9 kg at birth, with APGAR (Appearance, Pulse, Grimace, Activity, and Respiration) scores of 7, 8, and 9 at the first, fifth, and tenth minutes respectively [4]. The cephalic circumference was 34 cm, chest diameter was 30 cm, heart rate was 156 beats per minute, and respiratory rate was 72 breaths per minute. The purpose of this case report was explained to the parents of the newborn and written informed consent was obtained from the parents before case reporting and image publication.

**Figure 1:**
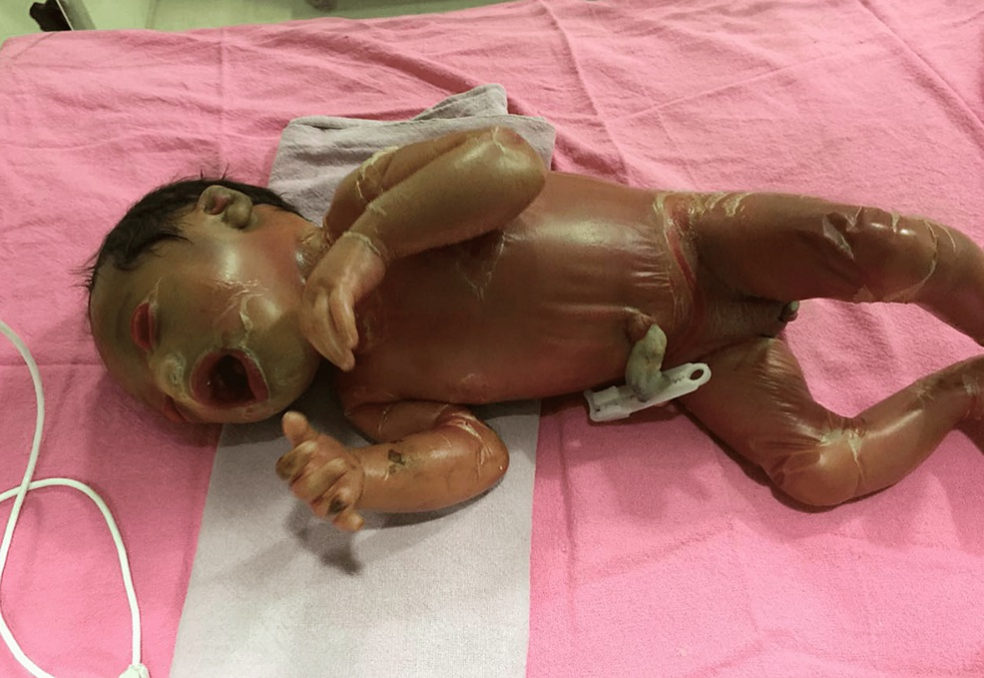
The collodion baby with generalized erythema and edema all over the body surface with ectropion and eclabium. Written informed consent was taken from parents before image publication

Upon examination, she was noted to have a generalized parchment-like membrane with crackling and peeling over the abdomen and extremities. Systemic findings revealed dehydration due to hyponatremia. Additionally, the other clinical findings demonstrated were ectropion (eversion of the eyelids), eclabium (eversion of the lips), flattening of the nose, and limited joint movement (Figure 1).

The anterior fontanelle was normal, air entry was bilaterally symmetrical in the lungs, and heart sounds were normal, without murmurs. The abdomen was soft and not distended, and genitalia was present normally in accordance with age and sex. The mother attended only five antenatal check-ups at the primary health center, although her anomaly scan was not suggestive of any abnormalities. There was no evidence of maternal complications or drug exposure during the parturition.

The management strategy focused on providing protection to the skin barrier, maintenance of fluid and electrolyte balance, and early initiation of retinoid therapy. The newborn was managed in the NICU in a strictly sterile environment to avoid the risk of infections, with adequate intravenous hydration and humidification in an incubator under the observation of a pediatrician. The newborn was kept in an incubator with humidity ranging from 50-60% as it decreases trans-epidermal water loss. Additionally, the temperature of the incubator was kept slightly lower (32-34°C) to maintain body temperature as impaired sweating can result in overheating.

The conventional skin management approach with topical emollients helped in softening of skin, which allowed appropriate movement and deeper respiration by the infant, followed by adequate lubrication of the exposed eye to prevent damage, and application of artificial tears and moisturizers was undertaken to prevent dryness of the eyes and skin. Furthermore, persistent ectropion was managed medically with periocular retinoids involving tazarotene as it accelerates the shedding of hyperkeratotic plates and improves scaling. Physiotherapeutic intervention was also performed to increase mobility and manage skin contractures. The newborn was discharged after she was stable and showed no signs of clinical complications. The parents were provided with appropriate counseling and instructions on childcare; however, they failed to follow up. 

## Discussion

The least frequently described form of congenital ichthyosis with a low prevalence is CB, which is secondary to disorders of cornification. It is predominantly inherited as autosomal recessive ichthyosis which may manifest as LI or NBCIE and presents with an underlying dermatological condition after the shedding of the collodion during the first to fourth week of life. Various studies corroborate that associated complications such as sepsis, hypothermia, severe erythroderma, dehydration, electrolyte imbalance, and constraining bands of the extremities leading to limited movement and edema of limbs can be managed through multidisciplinary supportive treatment care [5]. Expeditious correction of dehydration is necessary because such neonates are at an increased risk of electrolyte imbalance and fluid loss owing to impaired skin permeability function [6].

Kale et al. illustrated the importance of invasive prenatal tests and genetic diagnosis that may aid in the detection of the disorder [7]. The newborn in this case report was full-term at 38 weeks of gestation; however, due to fewer antenatal checkups attended by the mother, ultrasonography revealed no evident anomalies. Hence, a prenatal diagnosis could not be established owing to the lack of suspicion.

Ideally, such neonates should be admitted to the NICU and cared for in high-humidity incubators; however, this is challenging in resource-limited settings which may fail to provide the necessary interventions. Although a CB has lower odds of survival, previous studies have reported chances of survival beyond seven years of age. As proposed by Glick et al., this condition requires long hours of vigilance and appropriate multidisciplinary treatment care during the initial weeks of life [8].

## Conclusions

This case report proposes that despite the known complications associated with this rare condition, the early death of such neonates can be avoided by providing basic supportive neonatal care that includes a multidisciplinary treatment approach, regular antenatal checkups, and early prenatal diagnosis. The dynamic nature of this condition makes it challenging for clinicians to provide prompt care, which may hinder their chances of survival. Therefore, a suitable protocol must be devised to cater to such babies, with a focus on enhancing their quality of life. Initial assessments, such as genetic diagnosis and genetic counseling, must also be considered to improve the prognosis of this rare disorder.
